# The Plant Heat Stress Transcription Factors (HSFs): Structure, Regulation, and Function in Response to Abiotic Stresses

**DOI:** 10.3389/fpls.2016.00114

**Published:** 2016-02-09

**Authors:** Meng Guo, Jin-Hong Liu, Xiao Ma, De-Xu Luo, Zhen-Hui Gong, Ming-Hui Lu

**Affiliations:** ^1^Department of Vegetable Science, College of Horticulture, Northwest A&F UniversityYangling, China; ^2^Vegetable Research and Development Centre, Huaiyin Institute of Agricultural Sciences in Jiangsu Xuhuai RegionHuaian, China

**Keywords:** plant, heat stress, transcription factors, heat shock proteins, abiotic stress, transcriptional regulation

## Abstract

Abiotic stresses such as high temperature, salinity, and drought adversely affect the survival, growth, and reproduction of plants. Plants respond to such unfavorable changes through developmental, physiological, and biochemical ways, and these responses require expression of stress-responsive genes, which are regulated by a network of transcription factors (TFs), including heat stress transcription factors (HSFs). HSFs play a crucial role in plants response to several abiotic stresses by regulating the expression of stress-responsive genes, such as heat shock proteins (*Hsps*). In this review, we describe the conserved structure of plant HSFs, the identification of *HSF* gene families from various plant species, their expression profiling under abiotic stress conditions, regulation at different levels and function in abiotic stresses. Despite plant HSFs share highly conserved structure, their remarkable diversification across plants reflects their numerous functions as well as their integration into the complex stress signaling and response networks, which can be employed in crop improvement strategies via biotechnological intervention.

## Introduction

Plants as sessile organisms are routinely confronted by a variety of abiotic or biotic stresses, such as water deficiency, high salt, extreme temperatures, chemical pollutants, oxidative stress, nematodes, herbivores, and pathogens (Al-Whaibi, 2011). Especially, abiotic stress is the primary cause of crop loss worldwide, reducing crop productivity by an estimated 50% annually (Wang et al., 2004). Unlike animals, plants could not change their sites to escape from the unfavorable stresses, but have attained certain adaptations to these rapidly changing stresses during evolution, such as the dominance of sporophyte that encloses the sensitive gametophyte, the presence of leaf epidermis with stomata for gas exchange, the formation of stress resistant dormant organs, and the presence of conducting tissues in long-lived and big plants for long-distance nutrient and water transport (Baniwal et al., 2004; Al-Whaibi, 2011). A network of interconnected cellular stress response systems is a prerequisite for plant survival and productivity (Scharf et al., 2012), and their understanding is important for developing new methods to enhance plant stress tolerance.

A complex stress response network and a wide array of mechanisms for adapting to plants' changing environments at the physiological, biochemical, and molecular levels increase the tolerance to the stresses (Bartels and Sunkar, 2005; Zhou et al., 2009; Nakashima et al., 2012). The phytohormone abscisic acid (ABA) produced under abiotic stress conditions, induces leaf stomata closure and triggers the activation of many stress-related genes, thus playing a key role in responses to abiotic stress factors (Lata and Prasad, 2011). With the molecular techniques such as microarray analysis and large-scale transcriptome analysis, a large array of abiotic stress responsive genes has been identified in plants (Fowler and Thomashow, 2002; Nakashima et al., 2009). These genes not only play a role in the protection of the cells from stress by the production of important enzymes and metabolic proteins (functional proteins) but also in regulating signal transduction and gene expression in the stress response (regulatory proteins; Lata and Prasad, 2011; Nakashima et al., 2012). Among the regulatory proteins, transcription factors (TFs) play a crucial role in the conversion of stress signal perception to stress-responsive gene expression by interacting with *cis*-acting elements present in the promoter region of various target stress-responsive genes in the signal transduction processes, thus activating signaling cascade whole network of genes that act together in enhancing plant tolerance to the harsh environmental conditions (Akhtar et al., 2012). In plant genomes, ~7% of the coding sequences are assigned to TFs and many of these often belong to large gene families compared with animals and yeasts, such as the heat stress transcription factors (*HSFs*) family (Baniwal et al., 2004; Udvardi et al., 2007).

Plant *HSFs* are the terminal components of a signal transduction chain mediating the expression of genes responsive to various abiotic stresses (Nover et al., 2001). Many studies have reported on the central roles of *HSFs* in various abiotic stresses, including heat stress (HS) (Scharf et al., 2012), however, most analyses of *HSFs* function in stress responses examine individual stresses, not a combination of abiotic stress factors. In natural conditions, plants are routinely subjected to a combination of different abiotic stresses, such as the combination of drought, heat, and salinity stresses (Sewelam et al., 2014). The response of plants to a combination of different abiotic stresses cannot be directly extrapolated from the response of plants to each of the different stresses applied individually, therefore it is crucial to characterize the acclimation responses of plants to a combination of abiotic stresses and identify multiple stress responsive genes (Mittler, 2006; Colmenero-Flores and Rosales, 2014). Comprehensive characterization of multifunctional *HSFs* will provide the basis for investigating their functions in plant abiotic stress responses. In this review, the focus will be on the recent progress of the roles of *HSFs* in abiotic stress responses, with an emphasis on HS. In addition, recent advances in characterization of *HSFs* regulation will be also discussed.

## Structure and classification of plant HSFs

**Figure 1:**
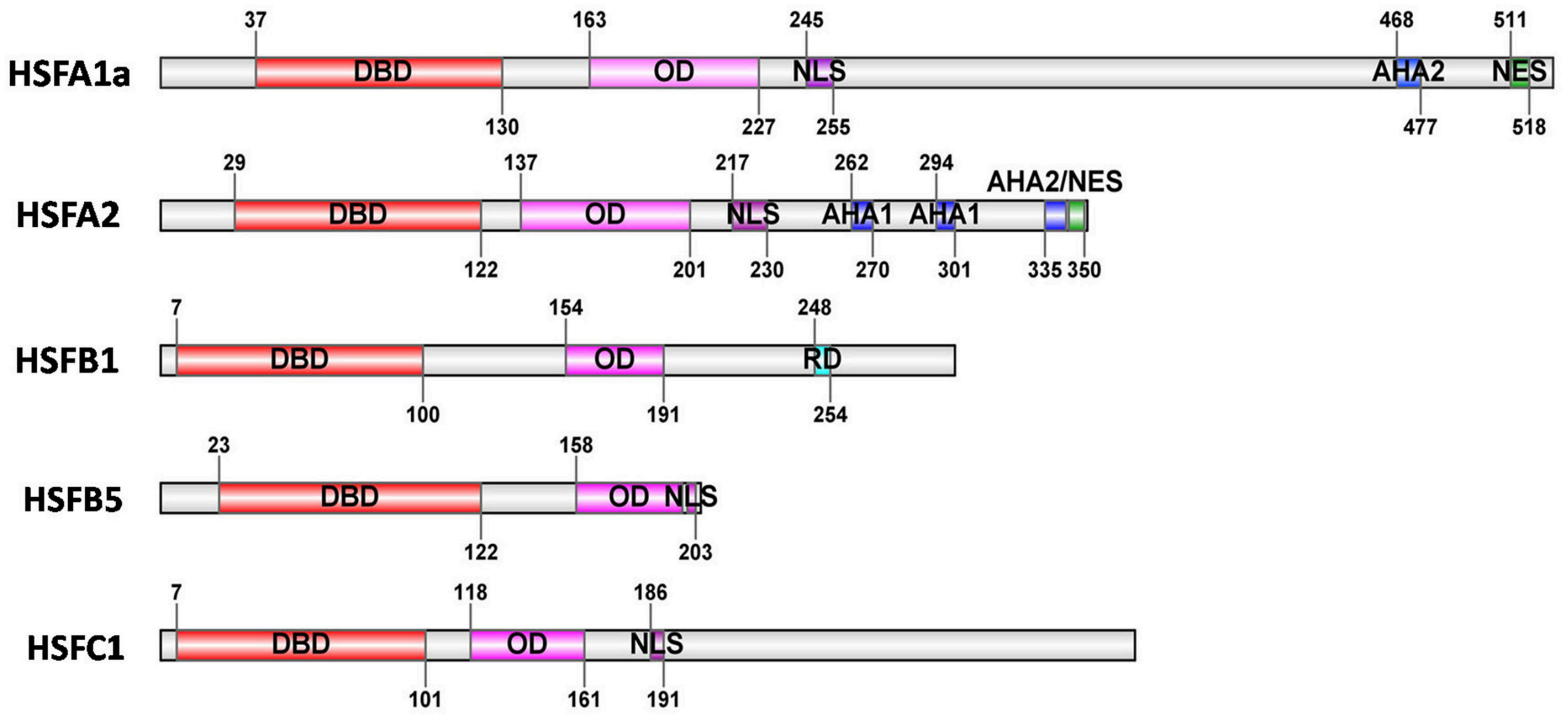
**Basic structure of HSFs**. The block diagrams represent five tomato HSFs with their conserved functional domains. The conserved domains are identified by Heatster (http://www.cibiv.at/services/hsf/). DBD, DNA binding domain; OD, oligomerization domain (HR-A/B region); NLS, nuclear localization signal; NES, nuclear export signal; AHA, activator motifs; RD, tetrapeptide motif–LFGV–as core of repressor domain. (*Adapted from* Scharf et al., 2012).

Typically, plant HSF proteins share a well conserved modular structure (Figure 1). The N-terminal DNA binding domain (DBD) is characterized by a central helix-turn-helix motif that specifically binds to the heat stress elements (HSEs) in the target promoters, and subsequently activates the transcription of stress-inducible genes (Baniwal et al., 2004; Sakurai and Enoki, 2010; Scharf et al., 2012). The oligomerization domain (OD) with a bipartite heptad pattern of hydrophobic amino acid residues (HR-A/B region) is connected to the DBD by a flexible linker (Baniwal et al., 2004). Based on the length of the flexible linker region between DBD and HR-A/B regions and the number of amino acid residues inserted into the HR-A/B regions, plant HSFs are classified into three classes, HSFA, B, and C (Nover et al., 2001; Kotak et al., 2004). The HR-A/B regions of HSFBs are compact and similar to all non-plant HSFs, however, members of class HSFA and C have an extended HR-A/B region due to an insertion of 21 (HSFAs) and 7 (HSFCs) amino acid residues between the HR-A and HR-B parts, respectively (Nover et al., 1996; Scharf et al., 2012). The C-terminal activation domains of plant HSFs are characterized by short peptide motifs (AHA motifs), which are crucial for the activator function in many cases (Döring et al., 2000). The AHA motifs formed of aromatic, large hydrophobic, and acidic amino acid residues, are HSFA-specific motifs but not found in class HSFB or C (Döring et al., 2000; Kotak et al., 2004). In addition, nuclear localization signal (NLS) and nuclear export signal (NES) of HSFs function in the assembly of a nuclear import complex built of the target protein and the receptor-mediated export in complex with the NES receptor exportin-α, respectively (Görlich and Kutay, 1999; Heerklotz et al., 2001; Baniwal et al., 2004). Notably, members of class HSFB (except HSFB5) comprise a characteristic tetrapeptide–**LFGV**–in the C-terminal domain, functioning as repressor domain (RD; Czarnecka-Verner et al., 2000; Ikeda and Ohme-Takagi, 2009; Fragkostefanakis et al., 2015).

## Identification of plant *HSF* families

Compared with few *HSF* members in vertebrates (4), *Drosophila* (1), *Caenorhabditis elegans* (1), and yeast (one HSF plus three HSF-related proteins; Nover et al., 1996; Nakai, 1999), plant *HSF* families comprise a large number of *HSF* members derived from a complex plant-specific superfamily and are present in a wide range of species. In the previous reports, the identification of the *HSF* family in plants was performed only in few model species such as *Arabidopsis*, tomato, and rice (Baniwal et al., 2004; Scharf et al., 2012). In recent years, based on the availability of an ever-increasing number of complete plant genomes and EST sequences, a large numbers of *HSF* families from more than 20 plant species have been identified at genome-wide scale. As shown in Table 1, there are 21 *HSF* encoding genes in *Arabidopsis* (Scharf et al., 2012), 24 in tomato (Scharf et al., 2012; Fragkostefanakis et al., 2015), 25 in pepper (Guo et al., 2015), 52 in soybean (Scharf et al., 2012), at least 56 in wheat (Xue et al., 2014), and so on. Compared with the *HSF* families of soybean, carrot (35 members) and cotton (40 members), the families of *Arabidopsis* and tomato are considered small. Currently, maximum of *HSF* genes were identified in wheat and soybean among monocots and eudicots, respectively. The multiplicity of *HSFs* in plants may be related to the gene duplications and whole-genome duplications at different points of evolution, followed by extensive gene loss (Scharf et al., 2012).

**Table 1:** **The *HSF* family in plant species**.

**Species**	**Number of *HSF* family**																	**References**
	***HSFA1***	***HSFA2***	***HSFA3***	***HSFA4***	***HSFA5***	***HSFA6***	***HSFA7***	***HSFA8***	***HSFA9***	***HSFB1***	***HSFB2***	***HSFB3***	***HSFB4***	***HSFB5***	***HSFC1***	***HSFC2***	**In total**	
*Arabidopsis thaliana*	4	1	1	2	1	2	2	1	1	1	2	1	1	0	1	0	21	Scharf et al., 2012
Tomato (*Solanum lycopersicum*)	4	1	1	3	1	2	1	1	1	1	2	2	2	1	1	0	24	Scharf et al., 2012; Fragkostefanakis et al., 2015
Castor bean (*Ricinus communis*)	2	1	1	2	1	1	1	1	1	1	2	1	2	1	1	0	19	Scharf et al., 2012
Pepper (*Capsicum annumm*)	3	1	1	3	1	3	0	1	4	1	2	2	1	1	1	0	25	Guo et al., 2015
Apple (*Malus domestica)*	4	2	3	1	2	0	0	2	2	2	1	2	2	0	2	0	25	Giorno et al., 2012
Tea (*Camellia sinensis*)	2	0	1	2	2	1	0	1	0	1	4	0	1	0	1	0	16	Liu et al., 2016
Soybean (*Glycine max*)	5	3	4	4	2	3	3	2	2	4	6	2	8	2	2	0	52	Scharf et al., 2012
Cotton (*Gossypium hirsutum*)	6	1	1	3	2	2	2	2	3	3	4	1	5	2	3	0	40	Wang et al., 2014
Chinese cabbage (*Brassica rapa pekinensis)*	8	1	1	1	1	4	2	1	0	2	3	2	2	0	2	0	30	Huang et al., 2015a
Poplar (*Populus trichocarpa*)	3	1	1	3	2	2	2	2	1	1	3	2	4	2	1	0	27	Scharf et al., 2012
Carrot (*Daucus carota*)	2	4	4	8	1	0	5	0	3	2	2	1	2	0	1	0	35	Huang et al., 2015b
strawberry (*Fragaria vesca)*	2	1	1	2	1	1	1	1	1	1	2	1	1	0	1	0	17	Hu et al., 2015
Willow (*Salix suchowensis)*	3	1	1	3	1	2	2	2	1	1	2	1	4	2	1	0	27	Zhang et al., 2015
Chinese white pear (*Pyrus bretschneideri*)	3	1	2	4	1	3	2	1	2	2	1	3	1	1	2	0	29	Qiao et al., 2015
Chinese plum (*Prunus salicina*)	2	1	1	2	1	1	1	1	1	1	2	0	1	1	1	0	17	Qiao et al., 2015
Peach (*Amygdalus persica*)	2	1	1	2	0	1	1	1	1	1	2	1	1	1	1	0	17	Qiao et al., 2015
European pear (*Pyrus communis*)	4	3	2	4	1	2	2	2	2	2	3	2	0	2	2	0	33	Qiao et al., 2015
Maize (*Zea mais*)	2	3	1	3	1	2	2	2	0	2	4	0	3	0	3	2	30	Scharf et al., 2012
Rice (*Oryza sativa*)	1	3	1	2	1	2	2	1	0	1	3	0	4	0	2	2	25	Scharf et al., 2012
Wheat (*Triticum aestivum*)	3	9	2	6	2	6	2	3	0	3	5	0	3	0	5	7	56	Xue et al., 2014
Millet (*Sorghum bicolor*)	1	3	1	2	1	2	2	1	0	1	3	0	3	0	2	2	24	Scharf et al., 2012
Brachypodium (*Brachypodium distachyon*)	1	3	1	2	1	2	2	1	0	1	3	0	3	0	2	2	24	Scharf et al., 2012

Interestingly, among the 25 species listed in Table 1, including 20 eudicots and 5 monocots, members of subclass *HSFA9, B3*, and *B5* were confined to the eudicots but not to the monocots, which emerged presumably after the split of monocots and eudicots. In addition, a variable number of the monocot-specific type *HSFC2* genes (2–7 genes) are found in all 5 monocots, not in eudicots, attributing to gene duplications on the monocot lineage. Higher number of class *HSFC* genes are identified in monocots, such as in wheat, maximum of 5 and 7 genes are assigned into subclass *HSFC1* and *C2*, respectively, which is the most marked difference between monocots and eudicots (Scharf et al., 2012). The large size of the plant *HSFs* family inevitable complicates the unraveling of their function under stress conditions.

## Expression analysis of plant *HSF* genes

The role of plant *HSFs* in abiotic stresses, especially in HS, has been recently brought to light (Fragkostefanakis et al., 2015). Although mRNA levels cannot be used to draw immediate conclusions about protein levels, they can point out directions of further investigations (Scharf et al., 2012). Genome-wide expression profiling of plant *HSF* genes under different abiotic stresses has been investigated extensively in various species. Most plant *HSFs* are regulated by HS, including up- and down-regulation. Upon HS, the transcript levels of *HSFA2* and *A6* members became the dominant *HSFs* in wheat, suggesting an important regulatory role during HS (Xue et al., 2014). Among 23 rice *OsHSF* genes, 16 *OsHSFs* were up-regulated by two-folds (log2 value) in response to HS, including 8 genes up-regulated by two-folds only during early heat shock (HS for 10 min) and 8 genes up-regulated at both short (HS for 10 min) and prolong (HS for 30 min) HS treatment, however, *OsHSFC1a* was noted to be down-regulated by the early HS treatment (Mittal et al., 2009), similarly, many *HSF* genes from different plant species, such as *GhHSF3, 18, 24, 32, 37*, and *40* from cotton (Wang et al., 2014), *ZmHSF-06*, -10, -14, -20, and -21 from maize (Lin et al., 2011), *MdHSFA9b* and *B4a/b* from apple (Giorno et al., 2012) showed down-regulation under HS treatment. The expression of *Arabidopsis HSFA2* was not detectable in control cell cultures but was detected strongly after HS treatment (Nover et al., 2001), and the similar situation also emerged in the expression profiles of pepper *CaHSFA2* (Guo et al., 2015), maize *ZmHSF-01* and *ZmHSF-04* (*HSFA2* group; Lin et al., 2011), apple *MdHSFA2a* and *A2b* (Giorno et al., 2012), and tomato *SlHSFA2* (Mishra et al., 2002). The HS-dependent translocation of *HSFA2* in *Arabidopsis* (Evrard et al., 2013) and tomato (Chan-Schaminet et al., 2009) and redox-dependent translocation of *AtHSFA8* (Giesguth et al., 2015) from the cytosol to nucleus may play central roles in plant HS and oxidative stress responses. In addition, many other abiotic stresses like cold, salinity and drought, and phytohormones such as jasmonic acid (JA), abscisic acid (ABA), salicylic acid (SA), and ethylene (Et) also have been shown to regulate the expression of plant *HSF* genes (Hu et al., 2015; Huang et al., 2015b; Zhang et al., 2015). The different abiotic stresses and phytohormone signaling pathways are assumed to interact and share some common elements that formed as potential “node” for crosstalk (Akhtar et al., 2012). These plant *HSF* genes may act as cross-point or node connecting several pathways and simultaneously regulate abiotic and phytohormone signaling pathways.

Plant *HSF* genes are not only induced by stress response but also by development, cell differentiation, and proliferation. For example, expression of *Arabidopsis AtHSFA2* gene increases during the process of callus formation and growth from root explants (Che et al., 2002). In addition, *HSFA2* is more highly induced in tomato anther than in the other flower tissues, and further induced under both short and prolonged HS conditions, which is similar to its expression in leaves (Giorno et al., 2010). In rice, the expression of *OsHSFA2a* gene is highly stimulated by HS particularly in root and shoot tissues as well as during panicle and seed development, while *OsHSFA7* and *A9* show developing seed-specific expression, in a similar pattern with those of *HSFA9* in sunflower and *Arabidopsis* (Chauhan et al., 2011; Scharf et al., 2012). These studies elaborate the border of conditions that are known to induce plant *HSFs* expression.

## Regulation of plant *HSF* genes

**Figure 2:**
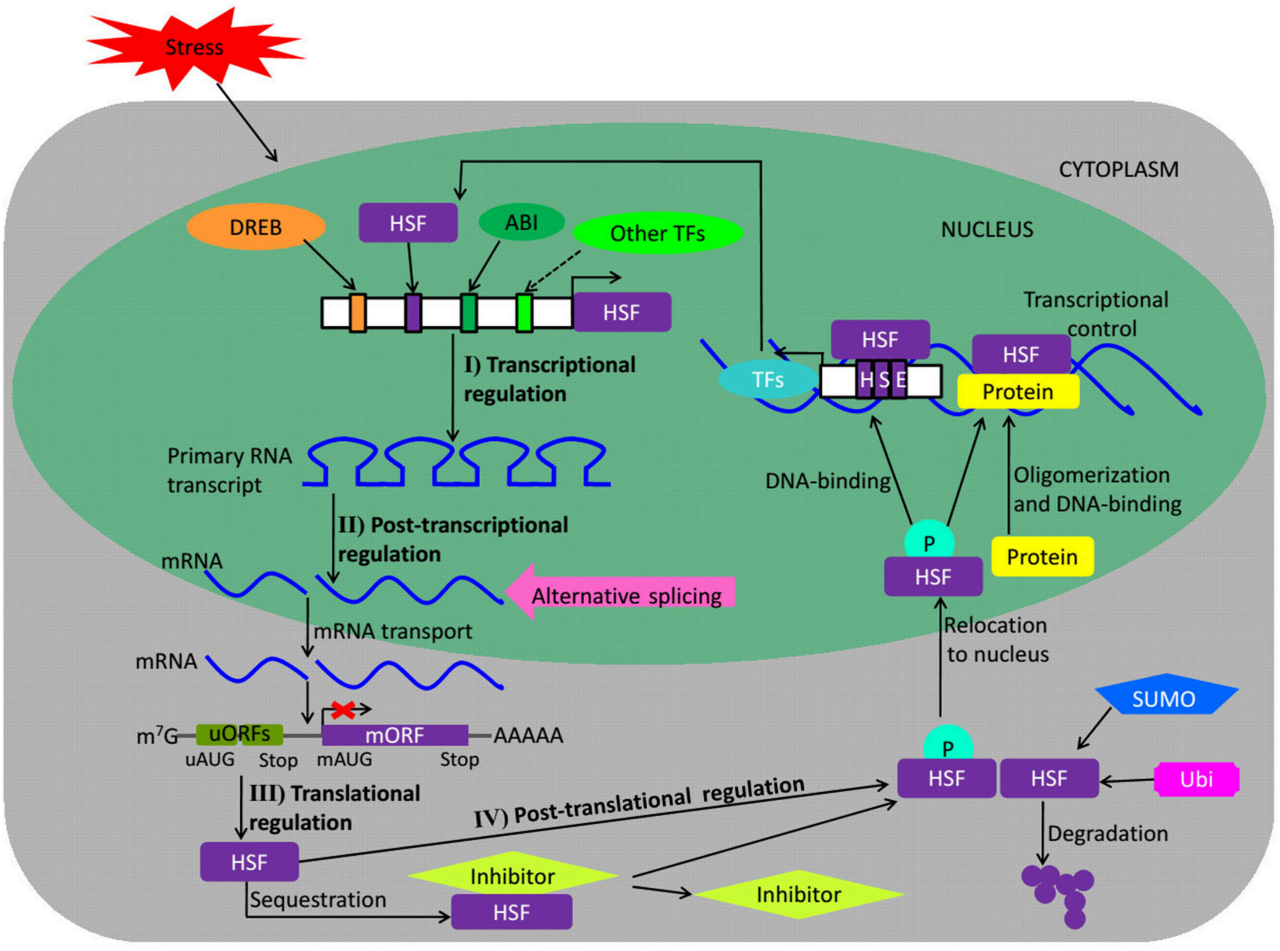
**Regulation of HSF proteins**. The scheme depicts the regulation of HSFs at different levels during stress. Upstream TFs like *DREB, HSF*, or *ABI* may bind to stress-related *cis*-regulatory elements in the promoter of regulated *HSF* genes and influence their transcription. Post-transcriptional control of *HSFs* by alternative splicing may also regulate their expression. The mature mRNAs are again governed during their transport and translation. uORFs regulate *HSFs* at the translation level. The translated protein may be subjected to activation by phosphorylation or undergo SUMO- and ubiquitin proteasomal-mediated degradation in response to certain environmental cues, other translated HSF proteins may be sequestrated by their inhibitors. Upon their nuclear import, the activated HSF proteins homo- or heterodimerize or bind to promoters of their target genes to control their expression. Broken arrows indicate possible but not firmly demonstrated routes. The red X mark represents translational repression. DREB, dehydration responsive element binding protein; ABI, ABSCISIC ACID–INSENSITIVE protein; TFs, transcription factors; AS, alternate splicing; mRNA, messenger RNA; m^7^G, cap of mRNA; uORFs, upstream micro open reading frames; mORF, major ORF; uAUG, AUG of uORF; mAUG, AUG of mORF; P, phosphate; SUMO, small ubiquitin-like modifier; Ubi, ubiquitination; HSE, heat stress element. (*Adapted from* Calkhoven and Ab, 1996; Puranik et al., 2012).

The studies on regulation of plant *HSFs* mainly focus on four levels including transcriptional, post-transcriptional, translational, and post-translation level (Fragkostefanakis et al., 2015). Transcription is the first step at which activity of a gene can be regulated by binding of specific TFs to the *cis*-acting elements located on the regulatory region of its promoter (Figure 2). The *Arabidopsis* AtHSFA1d and A1e binding to the HSE cluster in the 5′-flanking region of *AtHSFA2* gene is involved in high light (HL)-inducible *HSFA2* expression, activating *AtHSFA2* transcription (Nishizawa-Yokoi et al., 2011). Under HS, the *Arabidopsis* dehydration-responsive element (DRE)-binding protein 2A (*DREB2A* gene) directly regulates *AtHSFA3* transcription via binding the two DRE core elements in the *AtHSFA3* promoter (Yoshida et al., 2008). As *AtHSFA9* is exclusively expressed in late stages of seed development among the *Arabidopsis* family of 21 *HSFs*, a TF may be involved in the regulation of *AtHSFA9* expression during seed development. Kotak et al. (2007) reported that ABSCISIC ACID–INSENSITIVE3 (*ABI3* gene) could activate the *AtHSFA9* promoter based on an RY/Sph motif (8-bp sequence, **CATGCATG**) as putative seed-related regulatory element in the *AtHSFA9* promoter provided an essential binding site for ABI3. Interestingly, unlike *Arabidopsis AtHSFA1d* and *A1e, AtHSFB1* and *B2b* are transcriptional repressors and negatively regulate the expression of HS-inducible *HSFs* including not only *AtHSFA2* and *A7a* but also themselves (Ikeda et al., 2011).

Alternative splicing is a widespread process in eukaryotes that generates two or more different transcripts from the same precursor mRNA molecule by using different splice sites (Guerra et al., 2015). The complex post-transcriptional regulation of HSFs involves alternative splicing during different biological processes (Fragkostefanakis et al., 2015). Alternative splicing induced by HS is observed for *AtHSFA2, A4c, A7b, B1*, and *B2b* in *Arabidopsis. Arabidopsis AtHSFA2* derives from splicing of the conserved intron in the DBD, and a new heat stress-induced splice variant, *AtHSFA2-III* encodes a small truncated *AtHSFA2* isoform (*S-AtHSFA2*), which can bind to the TATA box-proximal clusters of HSE in the *AtHSFA2* promoter to activate its own transcription, attributing to exon skipping in the intron of the DBD encoding region (Sugio et al., 2009; Liu et al., 2013). The exon skipping pattern of *Physcomitrella patens PpHSFA1-1* is similar to that of *AtHSFA2*, which reveals that heat regulation for alternative splicing evolved early during land colonization of green plants (Chang et al., 2014). The alternative splicing induced by HS is also observed for rice *OsHSFA2d*, which encodes two main splice variant proteins, OsHSFA2dI localized to the nucleus and OsHSFA2dII localized to the nucleus and cytoplasm, respectively. The transcriptionally inactive spliced form of *OsHSFA2d*, OsHSFA2dII, is the dominant under normal conditions; however, once the plant suffered from HS, *OsHSFA2d* is alternatively spliced into the transcriptionally active form, OsHSFA2dI, which participates in the HS response and the unfolded protein response by regulating expression of *OsBiP1* (Cheng et al., 2015). *Medicago sativa MsHSF1* is composed of four exons and three introns in the primary transcript and generates five splice transcript isoforms, including one spliced transcript *MsHSF1b* encoding an HSFA1 protein that can specifically bind to the HSEs *in vitro* and four low-abundant spliced transcripts carrying the premature termination codon (He et al., 2007). These results suggest that the regulation of plant *HSFs* at post-transcriptional level is diversified.

Recently investigation suggests that the regulation of plant *HSFs* at translational level is mainly controlled by upstream micro open reading frames (uORFs) in their 5′ untranslated regions (Figure 2; Jorgensen and Dorantes-Acosta, 2012; von Arnim et al., 2014; Fragkostefanakis et al., 2015). However, the information on uORFs of plant *HSFs* is mainly restricted to *Arabidopsis*. Zhu et al. (2012) reported that 7 members out of 21 *Arabidopsis HSFs* have at least one uORF, including *AtHSFA1d, A1e, A2, A4a, B1, B2b*, and *C1*, but only for the uORFs of *AtHSFB1* and *B2b* there have been provided experimental evidence. The translation of *AtHSFB1* is regulated by uORF2 but not by uORF1, whereas, neither uORFs of *AtHSFB2b* are involved in regulation of the main ORF translation. The uORF2 represses the translation of *AtHSFB1* under normal condition, but the repression is deregulated under HS. The *Arabidopsis HSF*-like transcription factor *TBF1*, a major molecular switch for plant growth-to-defense transition, also contains two uORFs in the 5′ untranslated region. Unlike *AtHSFB1*, both uORFs of *TBF1* have inhibitory effects on *TBF1* translation, with the effect of uORF2 epistatic to that of uORF1. Both uORFs contain four phenylalanine (Phe) residues, and Phe starvation is shown to alleviate translational repression by the uORFs. Once plants are suffered from pathogen challenge, the uncharged tRNA^Phe^ will temporary increase and the eukaryotic initiation factor 2α (eIF2α) phosphorylation will be triggered, which may facilitate ribosome reattachment to the *TBF1* translation start codon downstream of uORFs and release the inhibitory effects of uORFs to initiate *TBF1* translation (Jorgensen and Dorantes-Acosta, 2012; Pajerowska-Mukhtar et al., 2012). In general, not only abiotic but also biotic stresses are involved in the translational regulation of plant *HSFs* controlled by uORFs. However, the mechanism of plant *HSFs*' translational control via uORFs is still scarce and needs further investigation.

Plant HSFs also undergo intensive post-translational regulation included phosphorylation, ubiquitination, and Small Ubiquitin-like MOdifier (SUMO)-mediated degradation, oligomerization, and interaction with other non-HSF proteins (Figure 2; Scharf et al., 2012; Song et al., 2012). In *Arabidopsis*, the mitogen-activated protein kinase MAPK6 specifically targets the AtHSFA2, phosphorylates it on T249 and changes its intracellular localization under HS conditions (Evrard et al., 2013); AtHSFA4A interacts with the MAP kinases MPK3 and MPK6 and is phosphorylated *in vitro* on three distinct sites, and Ser-309 being the major phosphorylation site (Pérez-Salamó et al., 2014). Nishizawa-Yokoi et al. (2010) reported that AtHSFA2 was regulated by the accumulation of polyubiquitinated proteins generated by the inhibition of 26S proteasome and AtHsp90. AtSUMO1 physically interacts with AtHSFA2 at the main SUMOylation site Lys315, leading to the repression of its transcriptional activity and ultimately disrupting the acquired thermotolerance pattern in *Arabidopsis* (Cohen-Peer et al., 2010). In addition, *Arabidopsis* FK506-binding proteins (FKBPs), ROF1 (FKBP62), and ROF2 (FKBP65) (Meiri and Breiman, 2009; Meiri et al., 2010), HSF binding protein (*AtHSBP*; Satyal et al., 1998), and tomato *Hsp17.4-II* (Port et al., 2004) also act as negative regulators for *HSFA2* transcriptional activity. Unfortunately, few active regulation factors involved in *HSF* regulation are found to date.

## Function of plant *HSFs* in HS stress response

The major objective for agronomic research remains the enhancement of crop productivity under various abiotic stresses (Puranik et al., 2012). Among the major abiotic stresses, HS has an independent mode of action on the physiology and metabolism of plant cells, and has a negative effect on plant growth and development, which may lead to catastrophic loss of crop productivity and result in widespread famine (Bita and Gerats, 2013). To deal with the threat posed by HS, unraveling the independent action and biological consequences is important. Based on the role of central regulators of the HS response (Baniwal et al., 2004), plant *HSFs* may be used for gene manipulation, contriving tolerance to HS in crops, while characterization of the functional plant *HSFs* under HS condition is the precondition.

Based on the previous studies, most current information on plant *HSFs* function under HS condition is derived from *HSFA1* and *A2* in tomato and *Arabidopsis*. *HSFA1* subfamily is defined as a master regulator of HS responses. Tomato *HSFA1a* has a unique function as master regulator for acquired thermotolerance, and cannot be replaced by any other *HSFs* (Mishra et al., 2002). However, no comparable master regulator activity could be identified for any of the four *AtHSFA1* (*a, b, d*, and *e*) with single or multiple mutants, and the role of master regulator for thermotolerance is shared among the four paralogs due to functional redundancy (Table 2; Liu et al., 2011; Scharf et al., 2012; Fragkostefanakis et al., 2015). Over-expression of soybeans *GmHSFA1* can enhance the thermotolerance of transgenic soybeans possibly due to the activation under HS of downstream genes, such as *GmHsp70, GmHsp22*, and other *GmHsps* (Table 2; Zhu et al., 2006). Based on its overall sequence (at the protein level) similarity to *HSFA1*s from other plant species (especially the well-characterized *LpHSFA1*) and its constitutive expression pattern, *GmHSFA1* may be the best candidate of master regulator in soybeans, which needs to be confirmed by an antisense silencing study. *HSFA2* has been identified to be the dominant *HSF* in tomato and *Arabidopsis* based on its high activator potential for transcription of *Hsp* genes and the strong accumulation under conditions of long-term HS or repeated cycles of HS and recovery (Mishra et al., 2002; von Koskull-Döring et al., 2007). HSFA2 and A1 form heterodimers resulting in synergistic transcriptional activation of HS genes after HSFA2 is accumulated in the nucleus of cells (Chan-Schaminet et al., 2009). Localization of the tomato HSFA2 protein to the nucleus evidently required interaction with HSFA1, whereas *Arabidopsis* HSFA2 protein can localize to the nucleus without interacting with the HSFA1 protein (Scharf et al., 1998; Kotak et al., 2004). Over-expression of *Arabidopsis HSFA2* in the *HSFA1* quadruple knock-out (*hsfA1a, b, d, and e*) mutant improved the thermotolerance, suggesting that *HSFA2* can be active and functional in the absence of *HSFA1s* in *Arabidopsis*, and it is tempting to speculate that interactions between HSFA2 and other HSFs may exist in the quadruple knock-out mutants (Liu and Charng, 2013; Fragkostefanakis et al., 2015). Enhanced thermotolerance has also been obtained by ectopic expression of rice *HSFA2e* and lily *HSFA2* in *Arabidopsis* (Table 2; Yokotani et al., 2008; Xin et al., 2010). In addition to the effects of *HSFA1* and *A2* members on the thermotolerance level, several other *HSFA* genes also function in the plant thermotolerance. For example, improved thermotolerance is observed in wheat plants over-expressing wheat *TaHSFA6f*, which relies on the concerted action of target genes, including *TaHsps* (*TaHSP16.8, TaHSP17, TaHSP17.3*, and *TaHSP90.1-A1*), *TaRof1, galactinol synthase*, and *glutathione-S-transferase* (*GST*; Xue et al., 2015); ectopic expression of tomato *HSFA3* and wheat *HSF3* in *Arabidopsis* also enhance its thermotolerance (Li et al., 2013; Zhang et al., 2013).

**Table 2:** **Overview of plant *HSF* genotypes and corresponding stress responses**.

**Genotype**	**Gene**	**Source of gene**	**Stress responses**	**References**
**OVER-EXPRESSION**				
	*AtHSFA1*	*Arabidopsis*	Increased thermotolerance in transgenic *Arabidopsis*	Lee et al., 1995
	*AtHSFA1b*	*Arabidopsis*	Enhanced water productivity, resistance to drought in transgenic *Arabidopsis*	Bechtold et al., 2013
	*AtHSFA2*	*Arabidopsis*	Increased themotolerance, salt/osmotic stress tolerance, and enhanced callus growth of transgenic *Arabidopsis*	Ogawa et al., 2007
	*AtHSFA2*	*Arabidopsis*	Increased tolerance to combined environmental stresses (high-light and heat-shock stresses) in transgenic *Arabidopsis*	Nishizawa et al., 2006
	*AtHSFA2*	*Arabidopsis*	Enhanced anoxia tolerance in transgenic *Arabidopsis*	Banti et al., 2010
	*AtHSF3*	*Arabidopsis*	Conferred thermotolerance in transgenic *Arabidopsis*	Prändl et al., 1998
	*AtHSFB1*	*Arabidopsis*	Repressed expression of *HSFA2, HSFA7a, HSFB2b, Hsp15.7CI* under moderate heat conditions (28°C) in transgenic *Arabidopsis*	Ikeda et al., 2011
	*AtHSFB2a*	*Arabidopsis*	Reduced biomass production in the early phase of growth and damaged development of female gametophytes in transgenic *Arabidopsis*	Wunderlich et al., 2014
	*LlHSFA1*	*Lilium longiflorum*	Interaction with *LlHSFA2*, enhanced thermotolerance in transgenic *Arabidopsis*	Gong et al., 2014
	*LlHSFA2*	*Lilium longiflorum*	Improved thermotolerance in transgenic *Arabidopsis*	Xin et al., 2010
	*OsHSFA2e*	*Oryza sativa*	Enhanced thermotolerance and tolerance to high-salinity stress in transgenic *Arabidopsis*	Yokotani et al., 2008
	*GmHSFA1*	*Glycine max*	Enhanced thermotolerance in transgenic soybean	Zhu et al., 2006
	*BhHSF1*	*Boea hygrometrica*	Increased thermotolerance in transgenic *Arabidopsis* and tobaccos	Zhu et al., 2009
	*VpHSF1*	*Vitis pseudoreticulata*	Reduced the basal thermotolerance, increased acquired thermotolerance, reduced the tolerance to osmotic stress in transgenic tobacco	Peng et al., 2013
	*VvHSFA9*	*Vitis vinifera*	Positive modulation of seed germination and might negatively regulate flowering time of transgenic *Arabidopsis*	Li et al., 2015
	*SlHSFA1*	*Solanum lycopersicum*	Master regulator of thermotolerance in transgenic tomato	Mishra et al., 2002
	*SlHSFA3*	*Solanum lycopersicum*	Increased thermotolerance and salt hypersensitivity during seed germination in transgenic *Arabidopsis*	Li et al., 2013
	*TaHSF3*	*Triticum aestivum*	Enhanced tolerance to extreme temperatures in transgenic *Arabidopsis*	Zhang et al., 2013
	*TaHSFA4a*	*Triticum aestivum*	Enhanced Cd tolerance by upregulating metallothionein gene expression in rice plants	Shim et al., 2009
	*TaHSFA6f*	*Triticum aestivum*	Improved thermotolerance in transgenic wheat	Xue et al., 2015
	*CarHSFB2*	*Cicer arietinum*	Increased tolerance to drought and heat stress in transgenic *Arabidopsis*	Ma et al., 2016
	*HaHSFA4a and A9*	*Helianthus annuus*	Synergistic functional effected on tolerance to severe dehydration and to drastic oxidative stress in transgenic tobacco	Personat et al., 2014
**MUTANT**				
	*AtHSF1 and AtHSF3*	*Arabidopsis*	No obvious effects on the heat shock response in the individual mutant lines; double mutants were significantly impaired in HS gene expression	Lohmann et al., 2004
	*AtHSFA2*	*Arabidopsis*	The expression of *AtHSFA2* was strictly heat stress-dependent and this transcription factor represented a regulator of a subset of stress response genes (*Hsp26.5, Hsp25.3, Hsp70b, APX2, RD29A, RD17, GolS1, IPS2, KSC1, ERD7, and ZAT10*) in *Arabidopsis*	Schramm et al., 2006
	*AtHSFA2*	*Arabidopsis*	*AtHSFA2* knockout mutant showed an obvious phenotype, and was more sensitive to severe HS than the wild type after long but not short recovery periods. Acquired thermotolerance (AT) decayed faster in the absence of *HSFA2*. *Hsa32* and class I small *Hsp* were less abundant in the mutant than in the wild type after long recovery. *AtHSFA2* sustained the expression of *Hsp* genes and extended the duration of AT in *Arabidopsis*	Charng et al., 2007
	*AtHSFA2*	*Arabidopsis*	Heat-dependent acclimation to anoxia was lost in an *HSFA2* knockout mutant	Banti et al., 2010
	*AtHSFB2a*	*Arabidopsis*	Knockdown of *asHSFB2a* correlated with an improved biomass production early in vegetative development but with an impaired development of female gametophytes	Wunderlich et al., 2014
	*AtHSFA1a/A1b/A1d/ A1e*	*Arabidopsis*	Members of the *AtHSFA1* group not only played a pivotal role in HSR but also were involved in growth and development. The basal and acquired thermotolerance capacity was dramatically decreased in the *QK* mutant but varied in triple KO mutants at different developmental stages. Increased sensitive phenotype of the *QK* mutant to H_2_O_2_, salt and mannitol stresses	Liu et al., 2011
	*AtHSFA1a/A1b/A1d/ A1e*	*Arabidopsis*	Constitutive expression of *AtHSFA2* rescued the developmental defects of the QK mutant and promoted callus formation in A2QK, but not in A2Wt, after heat treatment. Ectopic expression of *AtHSFA2* complemented the defects of QK in tolerance to different heat stress regimes, and to hydrogen peroxide, but not to salt and osmotic stresses, which revealed the overlapping and distinct functions of class *A1* and *A2 HSFs* in *Arabidopsis*	Liu et al., 2013
	*AtHSFA1d* and *A1e*	*Arabidopsis*	Double knockout mutant significantly suppressed the induction of *HSFA2* expression in response to HL and heat shock (HS) stress; *HSFA7a, A7b, B1*, and *B2a* were down-regulated compared with those in the wild-type plants under HL stress. The PSII activity of double mutants decreased under HL stress, and double knockout impaired tolerance to HS stress	Nishizawa-Yokoi et al., 2011
	*AtHSFB1* and *B2b*	*Arabidopsis*	In double mutant plants, the expression of a large number of heat-inducible genes was enhanced in the non-heat condition (23°C) and the plants exhibited slightly higher heat tolerance at 42°C than the wild type; expression of the heat-inducible *HSF* genes remained consistently higher in mutant than in the wild type under extended heat stress conditions. *HSFB1* and *B2b* appeared to be necessary for the expression of heat stress-inducible heat shock protein genes under heat stress conditions, which was necessary for acquired thermotolerance	Ikeda et al., 2011
	*OsHSFA4a*	*Oryza sativa*	Cd tolerance was decreased in rice plants with knocked-down expression of *OsHSFA4a*	Shim et al., 2009

At, Arabidopsis thaliana; Ll, Lilium longiflorum; Os, Oryza sativa; Gm, Glycine max; Bh, Boea hygrometrica; Vp, Vitis pseudoreticulata; Vv, Vitis vinifera; Sl, Solanum lycopersicum; Ta, Triticum aestivum; Car, Cicer arietinum; Ha, Helianthus annuus; HSR, heat shock response; Wt, wild type; KO, knock-out; QK, quadruple KO; HL, high light; Cd, cadmium; asHSFB2a, a natural long non-coding antisense RNA; APX2, ascorbate peroxidase 2; RD29A and RD17, cold- and drought-regulated genes; GolS1, a galactinol synthase; IPS2, a myo-inositol-1-phosphate synthase; KSC1, a ketoacyl-synthase; ERD7, an ethylene responsive protein; ZAT10, a salt tolerance zinc finger transcription factor.

In contrast to *HSFAs, HSFBs* have no transcriptional activity on their own due to lack of an activator domain. The HS-induced tomato *HSFB1* was suggested to be coactivator of *HSFA1a* by assembling into an enhanceosome-like complex resulting in the strong synergistic activation of reporter gene expression (Fragkostefanakis et al., 2015). The coactivator function of *HSFB1* depends on the recruitment of the plant CREB binding protein (CBP) ortholog histone acetyl transferase *HAC1* (von Koskull-Döring et al., 2007). Tomato HSFA1a, A2, and B1 form a triad of functionally interacting HSFs that is responsible for the transcriptional level of HS responsive genes during plant HS response and recovery (Perez et al., 2009; Scharf et al., 2012). However, HSFB1 from *Arabidopsis* was inactive as coactivator due to the essential histone-like motif **GRG****K****MMK** with an invariant Lys residue (underlined) in tomato HSFB1 is replaced by **GSRMTETK** in *Arabidopsis* HSFB1 (Bharti et al., 2004). Interestingly, *HSFB1* from *Arabidopsis* is characterized as a repressor of HS-inducible *HSFs*, such as *HSFA2, A7a, B1*, and *B2b*, however, the *hsfb1, hsfb2b* knockout mutant plants exhibit lower acquired thermotolerance than the wild type. This suggests that *HSFB1* and *HSFB2b* may promote the activity of *HSFA1* under HS conditions by repressing *Hsps* that interfere with the nuclear migration of *HSFA1s*, an activator of the early HS response (Ikeda et al., 2011). Over-expression of *VpHSF1* (a member of class *HSFB2* family) from Chinese Wild *Vitis pseudoreticulata* in tobacco demonstrated that *VpHSF1* acted as a negative regulator in basal thermotolerance and a positive regulator in acquired thermotolerance (Peng et al., 2013). The above results indicate striking species-specific deviation in the functional diversification of some members of the *HSF* family (von Koskull-Döring et al., 2007).

## Function of plant *HSFs* in other abiotic stress responses

Under natural conditions, plants frequently suffer from various abiotic stresses simultaneously; HS is compounded by additional abiotic stresses such as drought and salt stress (Bita and Gerats, 2013). The response of plant cells encountering a single stress condition can not reflect the real conditions in the field (Nishizawa et al., 2006). Gene manipulation of *HSFs* in plants is a significant approach to ameliorate the effects of combined HS and other abiotic stresses. Characterization of the functional *HSFs* involved in various abiotic stresses is necessary. The *Arabidopsis HSFA1s* are involved in response and tolerance to salt, osmotic, and oxidative stresses during seedling establishment (Liu et al., 2011). Especially, *Arabidopsis HSFA1b* controls a developmental component to drought tolerance and water productivity, however, the effect of *HSFA1b* over-expression on drought/dehydration tolerance does not involve changes in the expression of *DREB2A* or many other ABA- or dehydration-responsive genes (Bechtold et al., 2013). Given that *Arabidopsis HSFA3* is regulated by *DREB2A* as part of drought stress signaling pathway (Scharf et al., 2012), it is tempting to speculate that *Arabidopsis HSFA1b* and *A3* involve in different signal pathways to enhance the tolerance to drought stress. In addition, over-expression of chickpea *CarHSFB2* in *Arabidopsis* can increase the transcript levels of some stress-responsive genes (*RD22, RD26*, and *RD29A*) at seedling stage under drought stress conditions, thus improving their drought-tolerance (Ma et al., 2016); co-overexpression of sunflower *HaHSFA4a* and *A9* in transgenic tobacco results in synergistic effects on seedling tolerance to severe dehydration and oxidative stress (Personat et al., 2014). As the dominant *HSF* in thermotolerant cells, *HSFA2* also enhances tolerance to various other abiotic stresses, including salt/osmotic stress (Ogawa et al., 2007; Yokotani et al., 2008), anoxia stress (Banti et al., 2010), and combined high-light (HL) and HS stresses (Nishizawa et al., 2006). Unlike the above active regulation factors, tomato *SlHSFA3* and *V. pseudoreticulata VpHSF1* play negative roles in salt and osmotic stress, respectively (Li et al., 2013; Peng et al., 2013). These results suggest that the complex family of plant *HSFs* presents a functional diversity under different abiotic stress conditions.

## Conclusion and perspectives

**Figure 3:**
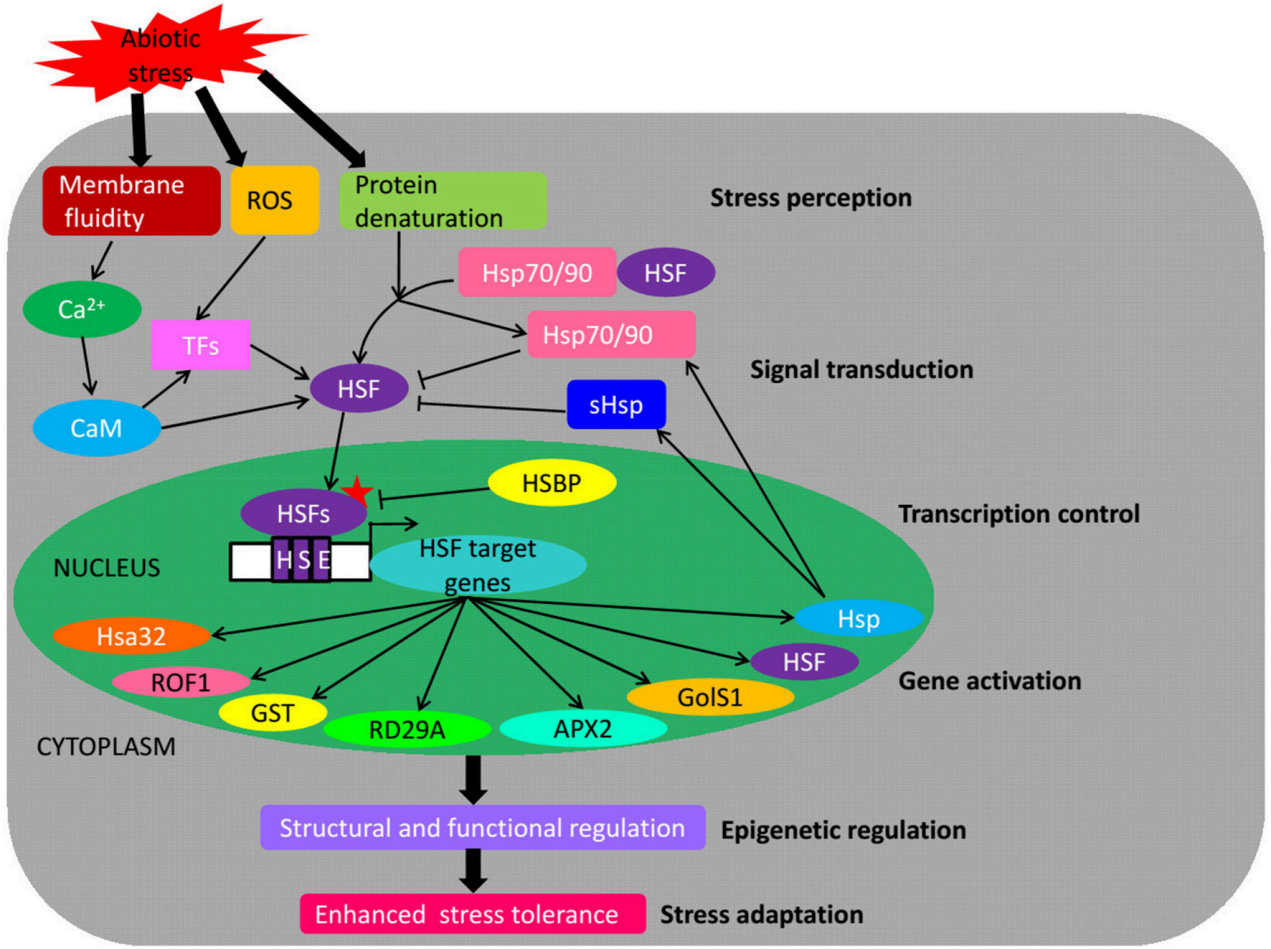
**Schematic representation of *HSFs* as key components in transcriptional regulatory networks during abiotic stress**. The scheme integrates both positive (arrows) and negative (bars) regulatory mechanisms. Abiotic stresses provoke a rise of cytoplasmic calcium, ROS accumulation and proteins denaturation inside the cells which convey stress-induced signals to responding genes, directly targeting HSF proteins marked with an asterisk. *HSFs* induce the activation of various genes playing a central role under abiotic stress conditions, thereby enhancing the abiotic stress tolerance. ROS, reactive oxygen species; CaM, Ca^2+^–calmodulin; TFs, transcription factors; Hsp, heat shock protein; sHsp, small Hsp; HSE, heat stress element; Hsa32, heat stress- associated 32-kD protein; Rof1, FK506-binding proteins; GST, glutathione-S-transferase; RD29A, drought-regulated gene 29A; APX2, ascorbate peroxidase 2; GolS1, a galactinol synthase; HSBP, HSF binding protein.

Understanding the molecular mechanisms of plants response to abiotic stresses such as heat, drought and salinity is a prerequisite for the manipulation of plants to improve stress tolerance and productivity. In response to these stresses, many genes are regulated mainly by TFs, and their gene products function in providing stress tolerance to plants (Lata and Prasad, 2011). One such class of the plant *TFs* is *HSF* that binds to HSE *cis*-acting elements in promoters of stress-inducible genes and plays central roles in the acquisition of plant tolerance against abiotic stresses. In this review, we have described the conserved structure of plant *HSFs*, the *HSF* gene families from various plant species based on the genome-wide identification, their expression profiling, different regulation levels and function in abiotic stresses. Plant *HSF* genes are important *TFs* that regulate the expression of various stress-responsive genes and play a key role in providing tolerance to multifarious abiotic stresses (Figure 3).

HSFs can be employed to engineer transgenic plants with higher tolerance to environmental stresses; however, many important questions should be addressed. The role of *HSF* genes in plants, especially in important agricultural crops needs a better understanding to minimize their negative effects in transgenic plants. For example, over-expressing *VpHSF1* in tobacco not only increased the acquired thermotolerance but also reduced the basal thermotolerance and the tolerance to osmotic stress (Table 2; Peng et al., 2013); over-expression of tomato *SlHSFA3* increased thermotolerance of transgenic *Arabidopsis*, but played a negative role in controlling seed germination under salt stress (Li et al., 2013). Because *HSFs* and chaperones play the broader role in cellular homeostasis, manipulation of *HSFs* may disrupt the homeostasis, leading to pleiotropic and undesired effects (Cabello et al., 2014; Fragkostefanakis et al., 2015). Although great progress has been achieved in the characterization of class *HSFA*s, the biological functions of *HSFB* and *C* members, and the *HSFs* active regulation factors remain to be clarified. Therefore, there is a dire need to understand the exact regulatory mechanisms of all the stress-responsive *HSF* genes. Most experiments on the role of *HSFs* in abiotic stress responses are limited to several model plants in laboratory conditions addressing individually abiotic stresses, which cannot represent precisely field conditions. As there is functional divergency between *HSF* orthologs in different plant species, it is necessary to adjust the research direction of *HSFs* function from few model plants to a broader variety of plant species, including the desired agricultural crops. In addition, marker-assisted selection can accelerate traditional crop breeding for stress tolerance traits, but decision of *HSFs* as candidate genes and developing proper functional markers has to be carefully decided due to the implication of *HSFs* in various developmental and stress response aspects (Fragkostefanakis et al., 2015).

In the future, a combination of advanced high throughput technologies, such as microarray, genomics, and proteomic approaches in various developmental stages and stress conditions will provide us with critical information to elucidate the whole complexity of *HSFs* integrated abiotic stress responses and different signaling pathways. Further studies are necessary to be focused on the functions of *HSFs* in agricultural crops under harsh field conditions, the dual (positive or negative) role of *HSFs* in different stress conditions and establishment of an HSF network in relation to the crosstalk between abiotic stress responses and plant growth, development and metabolism, which may provide practical and biotechnological approaches to improve the crop plants tolerance to extreme environment conditions.

## Author contributions

MG, ML, and ZG conceived and designed the paper; MG, JL, XM, and DL collected and analyzed the literature; MG wrote the paper.

### Conflict of interest statement

The authors declare that the research was conducted in the absence of any commercial or financial relationships that could be construed as a potential conflict of interest.
